# Decreased MiR-17 in glioma cells increased cell viability and migration by increasing the expression of Cyclin D1, p-Akt and Akt

**DOI:** 10.1371/journal.pone.0190515

**Published:** 2018-01-19

**Authors:** Guangwei Sun, Guozhong SiMa, Chunhui Wu, Yongzhong Fan, Yong Tan, Zhong Wang, Gang Cheng, Jie Li

**Affiliations:** 1 Department of Neurosurgery & Brain and Nerve Research Laboratory, The First Affiliated Hospital of Soochow University, Suzhou Shi, China; 2 Department of Neurosurgery, Danyang People’s Hospital, Danyang Shi, China; State University of New York, UNITED STATES

## Abstract

**Background:**

The activating mutations of micro RNA (miR)-17 have been revealed in tumors such as human non-Hodgkin's lymphoma and T cell leukemia. However, it is unclear about the role of miR-17 in glioma cells. The current study aimed to investigate effects of miR-17 mimics or inhibitor on the viability and migration of rat glioma C6 cells, and explore possible mechanisms.

**Methods:**

The expression of miR-17 in rat glioma C6 cells and normal brain tissue was detected by quantitative PCR. Protein expression of Cyclin D1 in rat glioma C6 cells and normal brain tissue was measured by Western Blot. Glioma C6 cells were transfected with MiR-17 mimics or inhibitor. Cells that were not transfected (Lipofectamine only) and cells that were transfected with nonsense RNA negative control served as control. MTT assay was utilized to detect cell viability, and cell wound scratch assay was utilized to examine the migration index. In addition, protein expression of Cyclin D1, p-Akt and Akt in MiR-17 mimics or inhibitor-transfected glioma C6 cells was detected by Western Blot. This study had been approved by the Medical Ethics Committee of the First Affiliated Hospital of Soochow University. All applicable international, national, and/or institutional guidelines for the care and use of animals were followed.

**Results:**

The expression of miR-17 was significantly lower, whereas the expression of Cyclin D1 was significantly higher in glioma C6 cells compared to normal brain tissue. MiR-17 mimics decreased the viability and migration of glioma C6 cells markedly at 48 h. In addition, MiR-17 inhibitor increased the viability and migration of glioma C6 cells at 24 and 48 h. The protein expression of Cyclin D1, p-Akt and Akt in glioma C6 cells decreased after transfection with miR-17 mimics for 72 h, and increased after transfection with miR-17 inhibitor for 72 h.

**Conclusions:**

The reduced miR-17 levels in glioma cells increased cell viability and migration, which correlates with increased expression of Cyclin D1, p-Akt and Akt.

## Introduction

Gliomas make up about 30% of tumors in brain and central nervous system and 80% of malignant brain tumors [1]. Gliomas are rarely curable, and the prognosis for patients with high-grade gliomas is poor, especially for elderly patients. The mortality rate of patients diagnosed with malignant gliomas was 50% after 1 year, and 25% after 2 years. Glioblastoma multiforme has a worse prognosis in gliomas, and the average survival time is within 1 year after diagnosis [2]. Therefore, it is of great importance to explore the characteristics and potential therapeutic targets of gliomas.

The miR-17 microRNA (miRNA) precursor family is a group of small non-coding RNA genes that regulate gene expression, and it includes miR-20a/b, miR-93 and miR-106a/b. MiR-17 miRNAs are produced from several miRNA gene clusters. The clusters are transcribed as long non-coding RNA transcripts and processed by Dicer enzyme to produce about 22 nucleotide products, which regulate gene expression by complementarity to the 3' UTR of target messenger RNA [3, 4]. The oncogenic potential of miR-17 gene clusters was first identified in mouse viral tumorigenesis screens [5]. The activating mutations of miR-17 were also revealed in human non-Hodgkin's lymphoma and T cell leukemia [6, 7]. In addition, miR-20a/b was reported to target the 3’ UTR of vascular endothelial growth factor (VEGF) and repress VEGF expression in nasopharyngeal carcinoma cell line [8]. Moreover, deletion of the miR-17 cluster has been shown to be lethal and result in developmental defects of lung and lymphoid cell in mice [9]. However, it is unclear about the role of miR-17 in glioma cells.

Cyclin D1 involves in the progression of cell cycle through G1 phase [10]. PI3K/Akt/mTOR pathway regulates cellular metabolism, growth and proliferation. Akt is an important component in PI3K/Akt/mTOR pathway, and it is a downstream effecter of PI3K. Akt is phosphorylated by its activating kinases, and phosphorylated Akt (p-Akt) are functional and active molecules that activate downstream signals of PI3K/Akt/mTOR pathway [11].

Therefore, we aimed to explore effects of miR-17 mimics or inhibitor on the viability and migration of rat glioma C6 cells, and investigate possible mechanisms by examining protein expression of cyclin D1, p-Akt and Akt in current study.

## Materials and methods

### Animals

Male Wistar rats were obtained from Shanghai SLAC Laboratory Animal Co. Ltd. C6 glioma cells in tumor-bearing rats were also purchased from Shanghai SLAC Laboratory Animal Co. Ltd., where DMEM culture-medium with 3×10^6^ C6 glioma cells was infused into rats to induce models of rats with tumor. They were fed with pelleted standard feed and water and were housed in steel cages at room temperature 23–26°C under 12/12 hours light dark cycle. All experiments were performed under sodium pentobarbital anesthesia. Animals are anesthetized by intraperitoneal injection with sodium pentobarbital solution (200mg sodium pentobarbital in 20ml of saline) at a dosage of 0.15mg/10g body weight and all efforts were made to minimize suffering. None of the rats showed any adverse effects or died before they were euthanized. All animal experiments were approved by the Department of Neurosurgery of Danyang People’s Hospital and were carried out in strict accordance with the recommendations in the Guide for the *Care and Use of Laboratory Animals of the National Institutes of Health*.

### Cells and reagents

Cells: Rat glioma C6 cell line (Chinese Academy of Sciences, Shanghai, China) were cultured at 37°C in Dulbecco’s modified eagle medium (DMEM) supplemented with 10% fetal bovine serum (FBS), 100 kU/L penicillin, and 100 mg/L chloramphenicol in a cell incubator with 5% CO_2_. The main reagents used in the studies and their suppliers were as follows: Trizol (Invitrogen Inc., Grand Island, NY, USA); miRNA reverse transcription kit (Tiangen Inc., Beijing, China); Takara reverse transcriptase M-MLV; common qPCR kit (Tiangen Inc., Beijing, China); RIPA tissue lysis buffer (Beyotime Inc; Shanghai, China); BCA protein quantification kit (Pierce Inc., Rockford, IL, USA); 30% acrylamide; Tris-HCl; sodium dodecyl sulfate (SDS), ammonium persulfate, tetramethylethylenediamine (TEMED); polyvinylidene fluoride (PVDF) membrane; phosphate buffered solution (PBS); Tween-20; electrochemiluminescence (ECL) solution; developing powder; fixing powder; X-ray films; inhibitor of micro-RNA-17); Trim8); specific primer for Cyclin D1 (Sangon Biotech Inc., Shanghai, China); primary antibody of β-actin (ZSGB-BIO Inc., Beijing, China); HRP-labeled goat anti-rabbit secondary antibody (Invitrogen Inc.; Grand Island, NY, USA); HRP-labeled goat anti-mouse secondary antibody (Beyotime Inc.; Shanghai, China).

The main pieces of equipment used in the study and the equipment suppliers were as follows: PCR machine Bio-Rad T-100; Step one Plus quantitative PCR machine (ThermoFisher Scientific Inc., Waltham, MA, USA); microplate reader (Kehua Inc.; Shanghai, China); micro-ultraviolet spectrophotometer (Nanodrop 2000); ice machine (Xuehua Inc., Changshu, China).

### Transfection of miRNA

After rat glioma C6 cells were cultured for 24 h, the original medium was changed into medium without serum or antibiotics. Mixture of Lipofectamine 2000 and miRNA was prepared; and negative control, miRNA mimic and miRNA inhibitors were diluted. The miR17 mimics and inhibitors purchased from GenePharm. Targeted miRNA sequences were shown as following: rno-miR-17-5p mimics: sense: 5’CAAAGUGCUUACAGUGCAGGUAG3’, antisense: 5’ACCUGCACUHUAAGCACUUUGUU3’, rno-miR-17-5p inhibitor: CUACCUGCACUGUAAGCACUUUG. Cells that were not transfected (treated with Lipofectamine only) and cells that were transfected with nonsense RNA negative control served as control. One hundred and twenty-five μL of OPTI-MEM was utilized to dilute RNA. One hundred and twenty-five μL of OPTI-MEM was also used to dilute 10 μL of Lipofectamine 2000, and incubated at room temperature for 5 min. The prepared RNA mixture and Lipofectamine 2000 mixture were then mixed, and incubated at room temperature for 20 min, which was added into antibiotic-free culture medium with glioma C6 cells. Culture medium was changed into antibiotic-containing medium after transfection of 4 to 8 h.

### MTT assay

After glioma C6 cells were transfected, they were cultured at 37°C and 5% CO_2_ for 24 h and 48 h, respectively. Twenty μL MTT (5 mg/mL) were then added into each well, and cultured for an additional 4 h. Following culture, the cell supernatants were removed and discarded, and 150 μL of DMSO was added to each well. The plates were then shaken for 15 min to dissolve crystals, and the absorbance of each sample was detected at 570 nm (A570) by an ELISA microplate reader. The degree of cell proliferation inhibition in each sample was calculated by the following formula: inhibition of cell proliferation (%) = (1 –absorbance of the experimental group/absorbance of the control group) × 100%.

### Cell wound scratch assay

Single cell suspension of rat glioma C6 cells in logarithmic growth phase was prepared, and cells were cultured in 6-well plates at 37°C and 5% CO_2_. Three horizontal lines were drawn onto the backs of 6-well plates using marker pens. Two hundred μL pipette tips were used to draw horizontal lines at the bottoms of the plates. The plates were then rinsed three times with PBS to eliminate cells that had peeled off when making the drawings. Glioma C6 cells were transfected and cultured at 37°C and 5% CO_2_ for 24 h and 48 h, respectively. Photos were taken at 0, 24, and 48 h after adding drug. The distance of migration and migration index were calculated by Image-Pro Plus 6.0 software (Media Cybernetics Inc., Rockville, MD, USA) and the following formula: inhibition of cell migration (%) = (1 –migration distance of the experimental group/migration distance of the control group) × 100%.

### RNA extraction

Fifty mg of rat brain tissue was added into 0.5 mL of Trizol, and homogenized on ice. The homogenate was transferred to Eppendorf tubes, and incubated at room temperature for 5 min. Chloroform (0.1 mL) was added into each tube, which was shaken for 15 sec and incubated at room temperature for 3 min. The tubes were then centrifuged at 12000 g and 4°C for 15 min. The supernatant was transferred into new Eppendorf tubes, and mixed with 0.25 mL of isopropanol. The mixture was incubated at room temperature for 10 min, and centrifuged at 12000 g and 4°C for 10 min. Supernatant was discarded, and 1 mL of 75% ethanol prepared with diethylpyrocarbonate (DEPC) -treated water was added into each tube. Tubes were vibrated and centrifuged at 7500 g and 4°C for 5 min. RNA was then dried and 20 to 50 μL of DEPC -treated water was added to each tube in order to dissolve RNA. Concentrations of RNA were then detected.

### Synthesis of first-strand cDNA

First-Strand miRNA was synthesized according to instructions of First-Strand Synthesis Kit (Tiangen Biotech Inc., Beijing, China). Poly A were added onto 3’ of miRNA by mixing total RNA, 0.4 μL of *E*. *coli* Poly(A) Polymerase, 2 μL of Poly(A) Polymerase, rATP Solution, and RNase-free ddH_2_O on ice. The tubes with the mixture were centrifuged shortly, and the mixture was then incubated at 37°C for 60 min. The reaction solution was utilized for experiments or stored in -20°C temporarily. cDNA was then produced by mixing poly(A) reaction solution, RT primer, RT buffer, super pure dNTPs, RNasin, Quant RTase, and RNase-free ddH_2_O. The mixture was centrifuged, and incubated at 37°C for 60 min. The synthesized cDNA was then utilized for experiments or stored in -20°C temporarily. Takara Reverse Transcriptase M-MLV was used to synthesize first-strand cDNA. Template RNA, primer, and RNase-free dH_2_O were mixed in a microtube, which was incubated at 70°C for 10 min and cooled on ice for 2 min. Reverse transcription solution was prepared in the above-mentioned microtube by mixing the prepared template and primer solution, M-MLV buffer, dNTP mixture, RNase inhibitor, RTase M-MLV, and RNase-free dH_2_O. The reverse transcription solution was then incubated at 42°C for 1 h, incubated at 70°C for 15 min, and cooled on ice. The derived cDNA solution could be used to synthesize second-strand cDNA directly or for PCR amplification.

### Quantitative fluorescence PCR (QF-PCR)

QF-PCR of miRNA was carried out according to instructions of QF-PCR kit (TIANGEN Biotech Inc., Beijing, China). MiRNA Premix and Reverse Primer were melted. MiRNA Premix, forward primer, reverse primer, miRNA first-chain cDNA, ROX reference dye, and ddH_2_O were mixed on ice. The mixture was pre-denatured at 94°C for 2 min. Then the mixture was denatured at 94°C for 20 sec, and DNA strands were annealed and extended at 60°C for 34 sec, which was repeated for 35 to 45 cycles.

For ordinary quantitative PCR, Real-PCR reaction system was established. Reagents, primers and template were melted. SuperReal Premix Plus, positive and negative primers, cDNA template, ROX reference dye, and RNase-free ddH_2_O were mixed on ice. The mixture was pre-denatured at 95°C for 15 min. Then the mixture was denatured at 95°C for 10 sec, and DNA strands were annealed and extended at 60–66°C for 20–32 sec, which was repeated for 40 cycles.

### Western Blot studies

C6 cells or brain tissue were rinsed with PBS. RIPA lysis buffer with inhibitors of protease and phosphatase were added into cell or tissue at 4°C. The solution was then centrifuged at 12000 rpm for 10 min, and supernatant was quantified and stored in -80°C fridge. Protein expression levels of Cyclin D1, p-AKT and AKT were detected by Western Blot. Cellular proteins were extracted and separated by electrophoresis (condensing gel: 120 V for 20 min; and separating gel: 180 V for 120 min) on a 10% SDS-polyacrylamide gel. The separated proteins were then electrophoretically (100 V for 120 min) transferred to a polyvinylidene fluoride (PVDF) membrane. After blocking with 5% non-fat milk powder for 1 h, the membranes were incubated with primary antibodies at 4°C overnight. Following incubation, the membranes were washed three times (10 min each) with solution of Tris-buffered saline and Tween 20 (TBST). The membranes were then incubated for one hour at room temperature with goat anti-rabbit secondary antibody labeled with horseradish peroxidase (HRP) (1/4000; Invitrogen Inc.; Grand Island, NY, USA). Membranes were washed and incubated for a short time period in electrochemiluminescence (ECL) solution. The films were exposed in a dark room. These experiments were repeated 3 times.

### Statistical analysis

The statistical data were analyzed and the figures were created using GraphPad Prism 5.0 software (GraphPad Software Inc.; La Jolla, CA, USA). All statistical results are expressed as the mean ± SEM. Differences between 2 groups were compared by student’s t test. Differences among 3 or more groups were compared by analysis of variance (ANOVA), followed by the Bonferroni post-hoc test for multiple comparisons. *P*-values ≤ 0.05 were considered statistically significant.

## Results

### Expression of miR-17 and Cyclin D1 in rat glioma C6 cells

**Fig 1:**
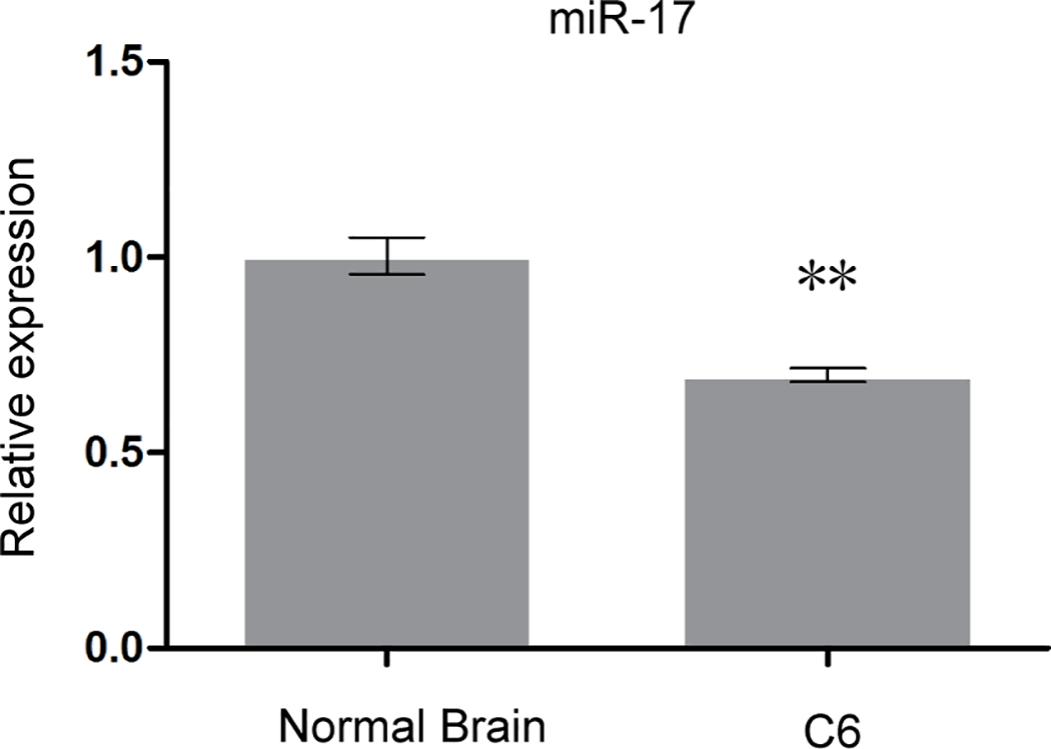
Relative expression of miR-17 in rat glioma C6 cells and normal brain tissue. The expression of miR-17 was detected by quantitative PCR. Results showed that the expression of miR-17 was significantly lower in rat glioma C6 cells compared to normal brain tissue. ** indicates that *p* < 0.01 when compared to normal brain tissue.

**Fig 2:**
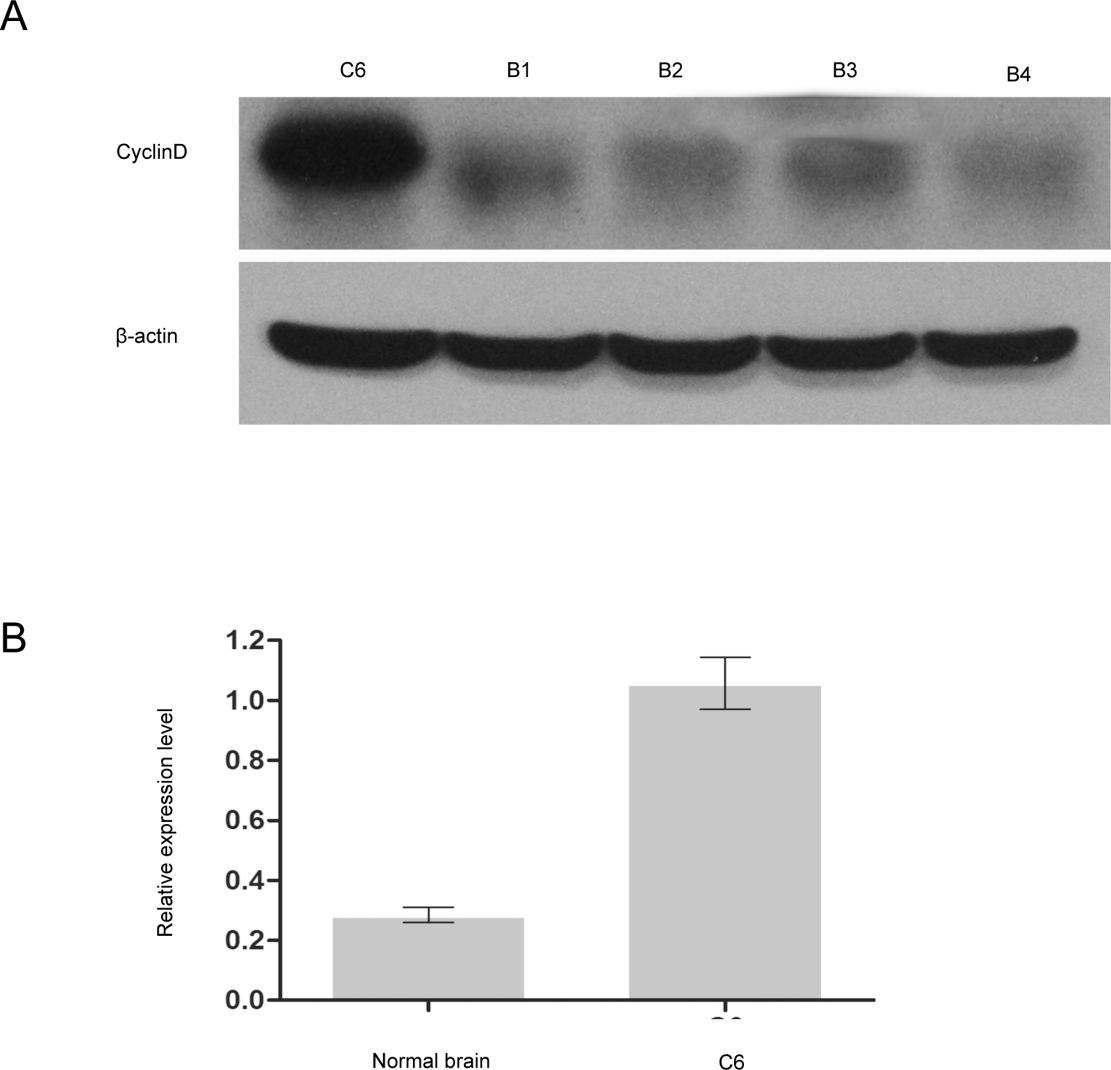
Protein expression of Cyclin D1 in rat glioma C6 cells and normal brain tissue. **(A)** Representative Western Blot image demonstrating the protein expression of Cyclin D1. **(B)** Quantification of Cyclin D1 expression. Results showed that the expression of Cyclin D1 was significantly higher in rat glioma C6 cells compared to normal brain tissue. *** indicates that *p* < 0.001 when compared to normal brain tissue.

The expression of miR-17 was detected by quantitative PCR, and expression of Cyclin D1 was examined by Western Blot. The expression of miR-17 was significantly lower (*p* < 0.01; Fig 1), and the protein expression of Cyclin D1 was markedly higher in rat glioma C6 cells compared to normal brain tissue (*p* < 0.001; Fig 2). The results indicated that miR-17 was negatively correlated with Cyclin D1.

### MiR-17 mimics decreased the viability and migration of glioma C6 cells

**Fig 3:**
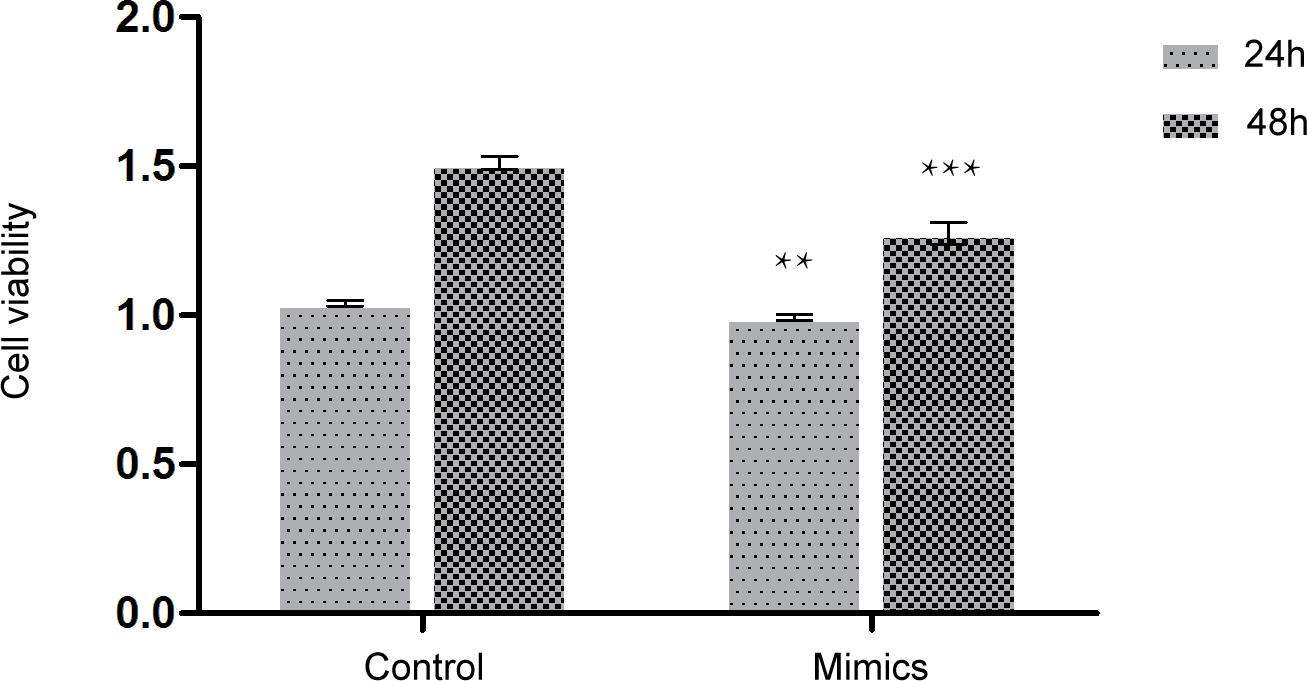
MiR-17 mimics decreased the viability of glioma C6 cells. MTT assay was utilized to detect the cell viability of glioma C6 cells. MiR-17 mimics (50 nM) decreased the viability of glioma C6 cells dramatically compared to Lipofectamine control group at 24 and 48 h. ** indicates *p* < 0.01, and *** shows *p* < 0.001 when compared to Lipofectamine control group. Lipo: Lipofectamine.

**Fig 4:**
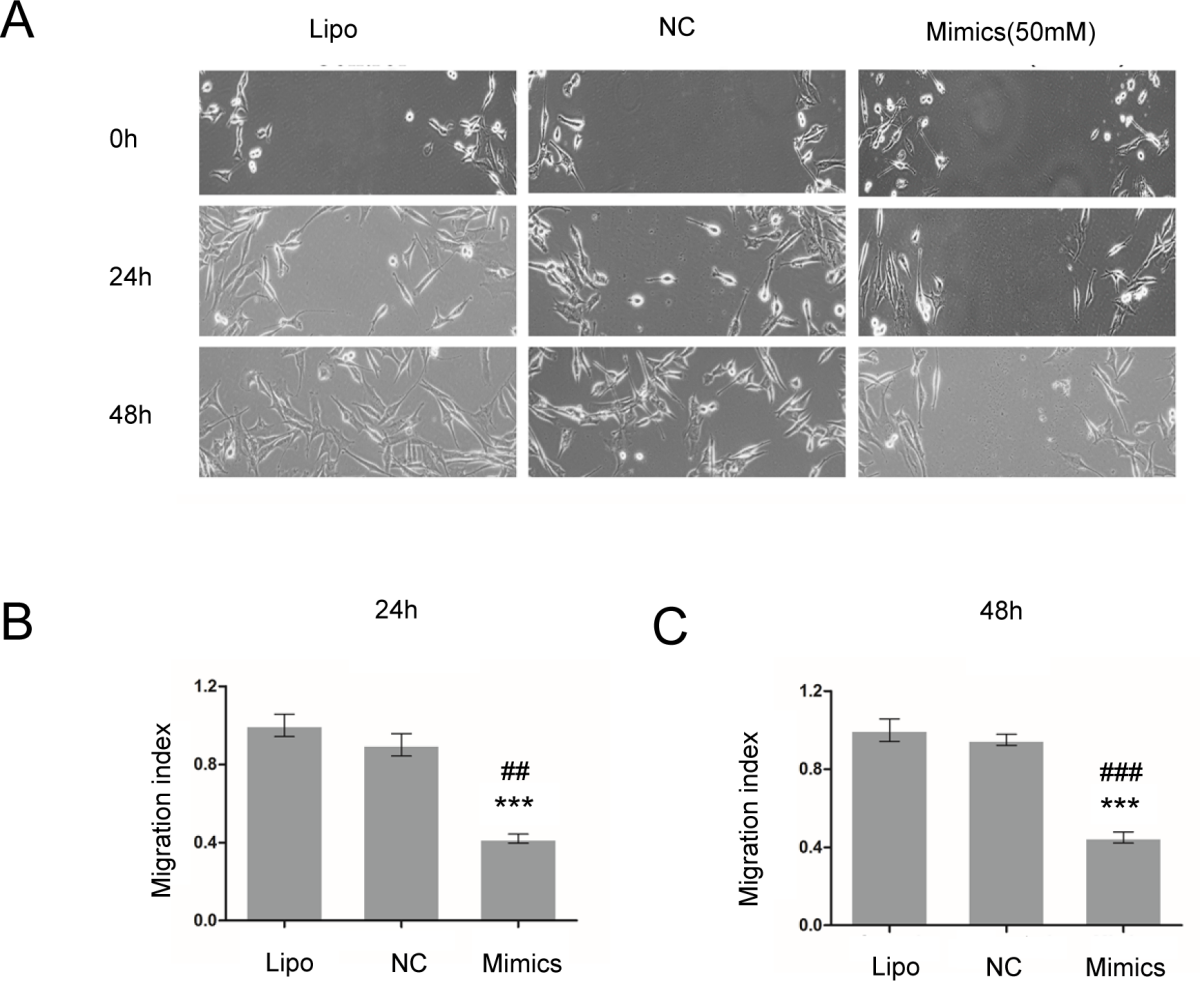
MiR-17 mimics decreased the migration of glioma C6 cells. Cell wound scratch assay was utilized to examine the migration index of glioma C6 cells. MiR-17 mimics (50 nM) decreased the migration of glioma C6 markedly compared to Lipofectamine and negative control groups at 48 h. *** shows that *p* < 0.001 as compared to Lipofectamine control group. ## indicates that *p* < 0.01 and ### demonstrates that *p* < 0.001 as compared to NC control group. Lipo: Lipofectamine. NC: negative control, cells transfected with nonsense RNA negative control.

MTT assay was utilized to detect the cell viability, and cell wound scratch assay was used to examine the migration index of glioma C6 cells. Relative cell viability in different groups to the control group at 24 and 48 h was presented in Fig 3. MiR-17 mimics (50 nM) significantly decreased the viability (24 h: 1.04 ± 0.0 versus 0.97 ± 0.0, *p* < 0.01; 48 h: 1.5 ± 0.0 versus 1.3 ± 0.0; *p* < 0.001; Fig 3) and migration (45.0 ± 2.9 versus 100.0 ± 5.8, *p* < 0.05; Fig 4) of glioma C6 cells compared to Lipofectamine control group at 48 h.

### MiR-17 inhibitor increased the viability and migration of glioma C6 cells

**Fig 5:**
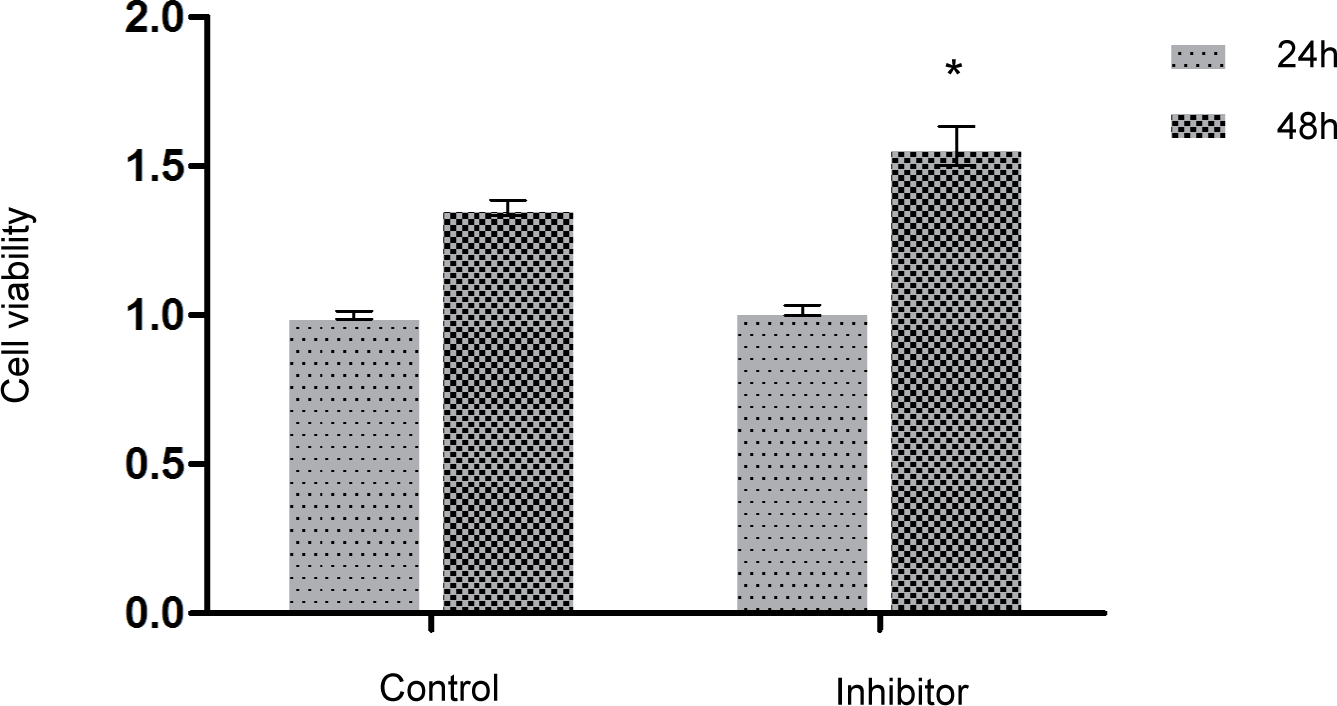
MiR-17 inhibitor increased the viability of glioma C6 cells. MTT assay was utilized to detect the cell viability of glioma C6 cells. MiR-17 inhibitor (200 nM) increased the viability of glioma C6 cells significantly compared to Lipofectamine control group at 48 h. * indicates that *p* < 0.05 when compared to Lipofectamine control group at 48 h. Lipo: Lipofectamine.

**Fig 6:**
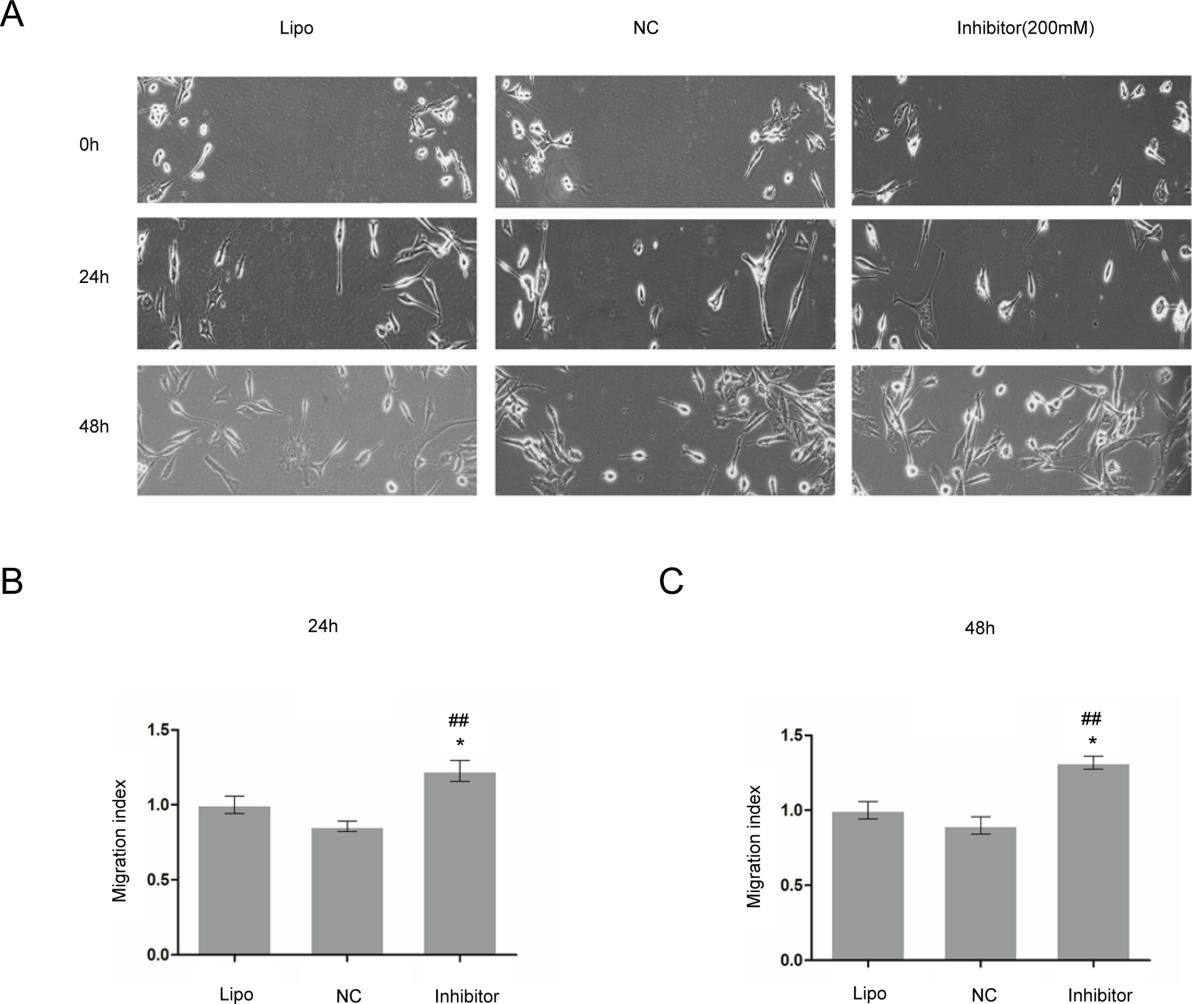
MiR-17 inhibitor increased the migration of glioma C6 cells. Cell wound scratch assay was utilized to examine the migration index of glioma C6 cells. MiR-17 inhibitor (200 nM) increased the migration of glioma C6 markedly compared to Lipofectamine and negative control groups at 24 and 48 h. * shows that *p* < 0.05 as compared to Lipofectamine control group; and ## indicates that *p* < 0.01 when compared to negative control group.

MTT assay was used to measure the cell viability, and cell wound scratch assay was used to detect the migration index of glioma C6 cells. Relative cell viability in different groups to the control group at 24 h was presented in Fig 5. MiR-17 inhibitor (200 nM) markedly increased the viability (1.5 ± 0.0 versus 1.4 ± 0.0, *p* < 0.05; Fig 5) and migration of glioma C6 cells (24 h: 0.86 ± 0.0 versus 1.2 ± 0.0, *p* < 0.05; 48 h: 0.90 ± 0.1 versus 1.3 ± 0.0, *p* < 0.05; Fig 6) compared to Lipofectamine control group.

### Protein expression of Cyclin D1, p-AKT and AKT decreased in miR-17 mimics-transfected glioma C6 cells

**Fig 7:**
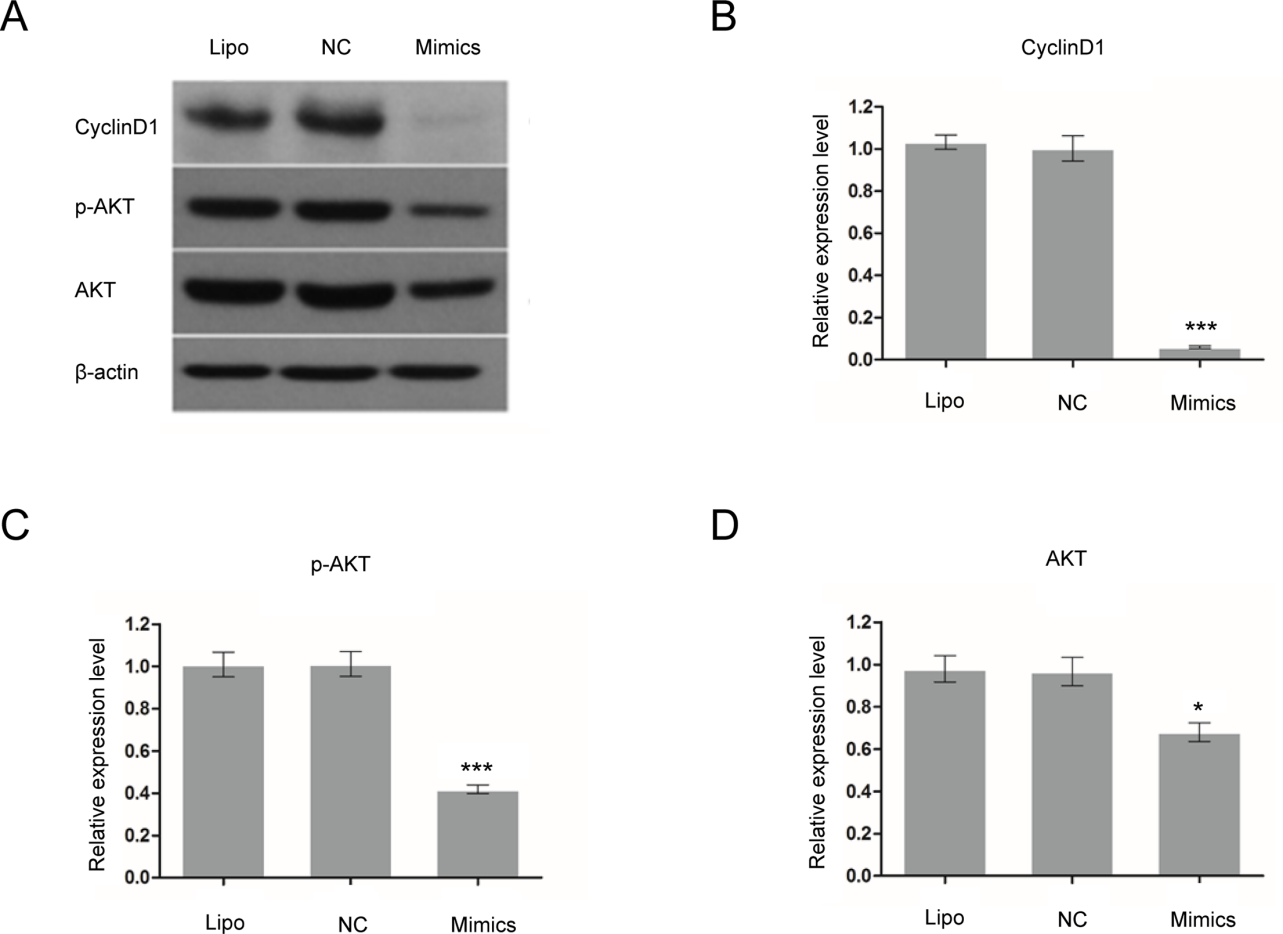
Protein expression of Cyclin D1, p-Akt and Akt decreased in miR-17 mimic-transfected rat glioma C6 cells. Protein expression of Cyclin D1, p-Akt and Akt in glioma C6 cells was detected by Western Blot. After glioma C6 cells were transfected withmiR-17 mimics (50 nM) for 72 h, the protein expression of Cyclin D1, Akt and p-Akt decreased compared to Lipofectamine and negative control groups. * indicates that *p* < 0.05; and *** shows that *p* < 0.001 when compared to Lipofectamine control group. Lipo: Lipofectamine. NC: negative control.

Protein expression of Cyclin D1, p-AKT and AKT in glioma C6 cells was detected by Western Blot. After glioma C6 cells were transfected withmiR-17 mimics (50 nM) for 72 h, the protein expression of Cyclin D1 (*p* < 0.001), p-AKT (*p* < 0.001) and AKT (*p* < 0.05) decreased compared to Lipofectamine and negative control groups (Fig 7). The relative protein expression of Cyclin D1, p-AKT and AKT in miR-17 mimic group to Lipofectamine control group was 5.8 ± 0.6%, 41.5 ± 2.1%, and 70.3 ± 4.4%, respectively.

### Protein expression of Cyclin D1, p-AKT and AKT increased in miR-17 inhibitor-transfected glioma C6 cells

**Fig 8:**
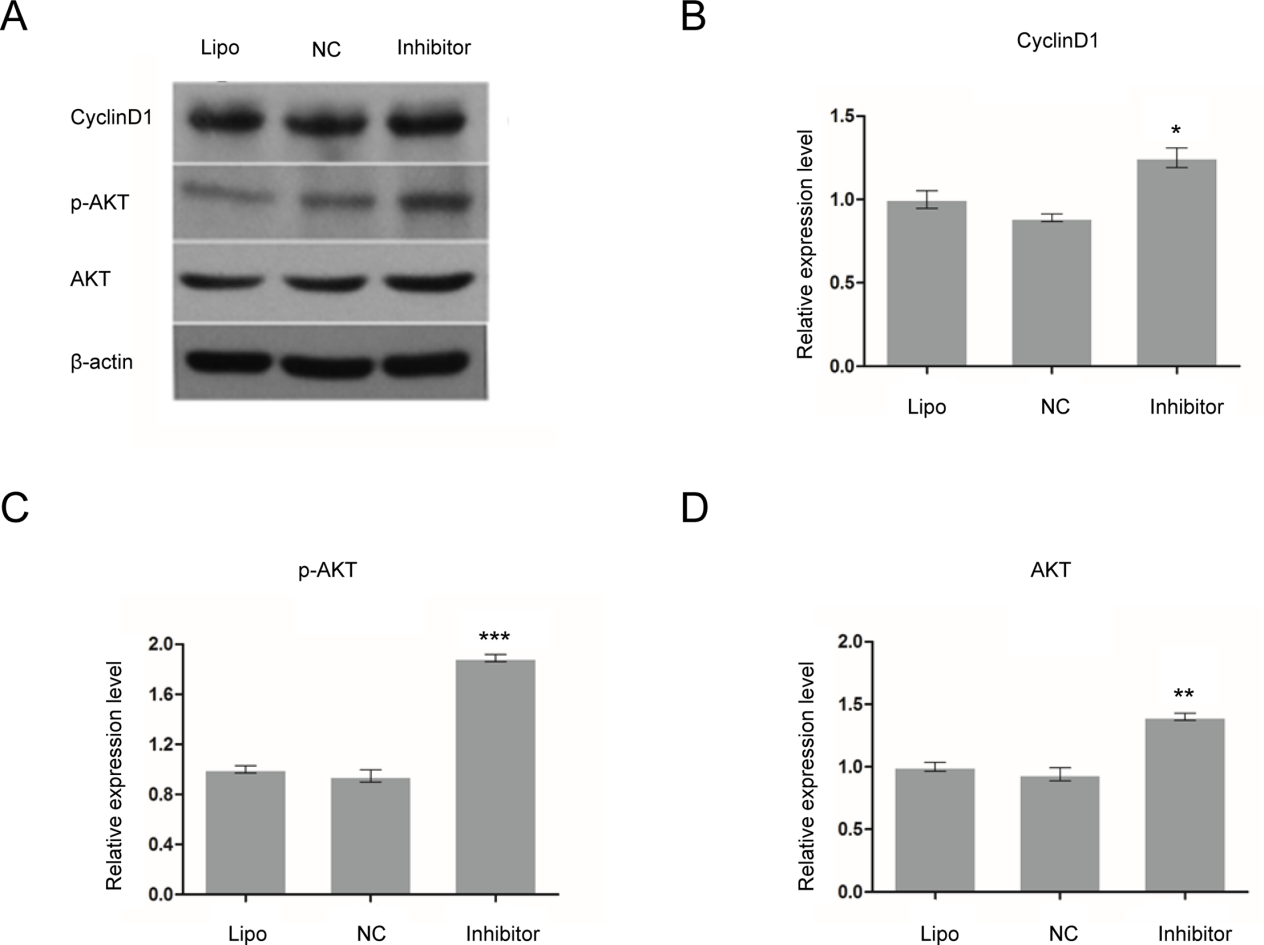
Protein expression of Cyclin D1, p-Akt and Akt increased in miR-17 mimic-transfected rat glioma C6 cells. Protein expression of Cyclin D1, p-Akt and Akt in glioma C6 cells was detected by Western Blot. After glioma C6 cells were transfected with inhibitor of miR-17 (200 nM) for 72 h, the protein expression of Cyclin D1, Akt and p-Akt increased compared to Lipofectamine and negative control groups. * indicates that *p* < 0.05; ** indicates that *p* < 0.01; and *** shows that *p* < 0.001 when compared to Lipofectamine control group. Lipo: Lipofectamine. NC: negative control.

Protein expression of Cyclin D1, p-AKT and AKT in rat glioma C6 cells was detected by Western Blot. After glioma C6 cells were transfected with inhibitor of miR-17 (200 nM) for 72 h, the protein expression of Cyclin D1 (*p* < 0.05), p-AKT (*p* < 0.001) and AKT (*p* < 0.01) increased compared to Lipofectamine and negative control groups (Fig 8). The relative protein expression of Cyclin D1, p-AKT and AKT in miR-17 inhibitor group to Lipofectamine control group was 1.3 ± 0.1, 1.9 ± 0.0, and 1.4 ± 0.0, respectively.

## Discussion

We have demonstrated that the expression of miR-17 is decreased in glioma cells, and inhibition of miR-17 increased the viability and migration of glioma cells. This correlates with increased expression of Cyclin D1, p-Akt and Akt.

MiRNAs are an extensive family of ∼22-nucleotide-long noncoding RNAs expressed in a wide range of eukaryotes including humans. Studies have revealed that miR-17 involves in several types of tumors, and effects of miR-17 on tumors vary depending on cells and tissue involved. MiR-17-5 was reported to have extensive complementarity to mRNA of Amplified In Breast Cancer 1 (AIB1). AIB1 expression was downregulated by miR-17-5p by translational inhibition, and the expression of miR-17-5p was low in breast cancer cell lines. MiR-17-5p also abrogated the insulin-like growth factor 1-mediated growth of breast cancer cells [12]. In addition, the genomic region encoding miR-17-92 cluster was shown to be amplified in lymphoma, and retroviral expression of miR-17-92 accelerated c-Myc-induced development of lymphoma [13]. Absence of miR-17-92 also resulted in increased pro-apoptotic protein Bim and inhibited B cell development at the transition from pro-B to pre-B [14].

In current study, we unveiled that inhibition of miR-17 increased the viability and migration of glioma cells. This indicated that miR-17 has a role as a tumor suppressor in glioma cells, and decreased miR-17 renders glioma cells unrestrained proliferation and metastasis. Lower level of miR-17 in gliomas may correlate with worse prognosis. We identify miR-17 as an attractive molecule that can possibly be used as a biomarker to diagnose gliomas early and predict the prognosis, and current study provides the rationale for therapeutic approaches to enhance miR-17 in glioma cells.

In addition, we revealed that decreased miR-17 in glioma cells correlated with increased expression of Cyclin D1. MiR-17 was shown to target the 3' UTR of Cyclin D1 gene in breast cancer cells with the highest score using bioinformatics analyses. The expression of miR-17 was decreased in breast cancer cell line, and gene expression of Cyclin D1 decreased after lentiviral transduction of miR-17 to breast cancer cells [15]. Therefore, it is possible that MiR-17 also targets the 3' UTR of Cyclin D1 gene in glioma cells, and decreased the protein expression of Cyclin D1 as shown in current study. This provides one explanation for the increased viability and migration in glioma cells with low expression of miR-17.

We also showed that low miR-17 levels in glioma cells correlated with increased expression of active p-Akt and Akt. The serine threonine kinase Akt has been implicated in controlling cellular survival, growth and metabolism, and Akt (-)/(-) mammary epithelial cells demonstrated increased apoptosis to DNA damaging agents. MiR-17/20 overexpression was reported to sensitize cells to apoptosis induced by either Doxorubicin or UV irradiation in breast cancer cells via Akt, and miR-17/20 mediated apoptosis via increased p53 expression which promoted the degradation of Akt [16]. Thus, miR-17 may degrade Akt by increasing the expression of p53 in gliomas. This provides another explanation for the increased viability and migration in glioma cells with low expression of miR-17.

In conclusion, we demonstrated for the first time that the low miR-17 levels in glioma cells increased cell viability and migration, which correlates with increased expression of Cyclin D1, p-Akt and Akt. Our results reveal that miR-17 has a role as a tumor suppressor in glioma cells. Therapeutic approaches to increase miR-17 levels may have potential in treating gliomas.

## Supporting information

S1 File Arrive checklist. (DOCX)
