# Supplementary material for: Decreased MiR-17 in glioma cells increased cell viability and migration by increasing the expression of Cyclin D1, p-Akt and Akt
Source: PLoS One. 2018 Jan 19;13(1):e0190515. doi: 10.1371/journal.pone.0190515 (PMC5774692; doi:10.1371/journal.pone.0190515)

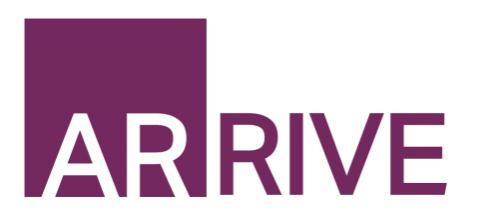


The ARRIVE Guidelines Checklist

Animal Research: Reporting In Vivo Experiments

Guangwei Sun^1,2#^, Guozhong SiMa ^2#^, Chunhui Wu ^2^, Yongzhong Fan ^2^, Yong Tan ^2^, Zhong Wang ^1*^, Gang Cheng ^1^, Jie Li ^2^

1. *Department of Neurosurgery & Brain and Nerve Research Laboratory, The First Affiliated Hospital of Soochow University*
2. *Department of Neurosurgery, Danyang People’s Hospital*
3. *# These authors contributed equally to this study and share first authroship.*
4. **Corresponding author*

|  | | ITEM | RECOMMENDATION | Section/ Paragraph |
| --- | --- | --- | --- | --- |
| 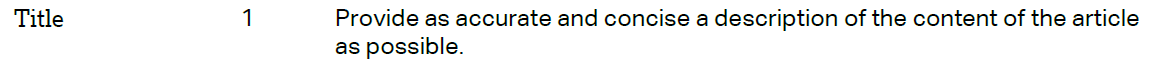 | | | Title |  |
| 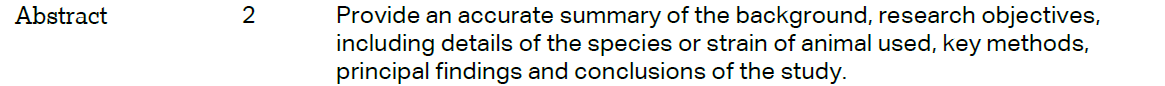 | | | Abstract |  |
| INTRODUCTION | | |  |  |
| 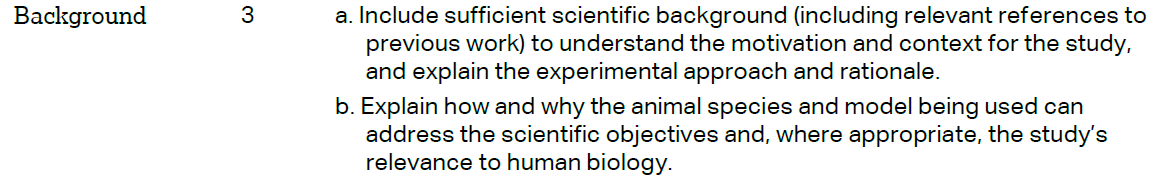 | | | Paragraphs 1-3  Paragraphs 2-3 |  |
| 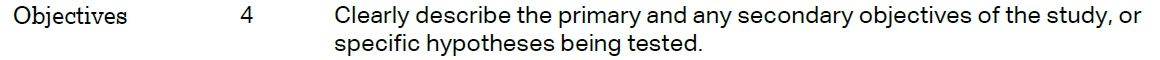 | | | Paragraph 3 |  |
| METHODS | | |  |  |
| 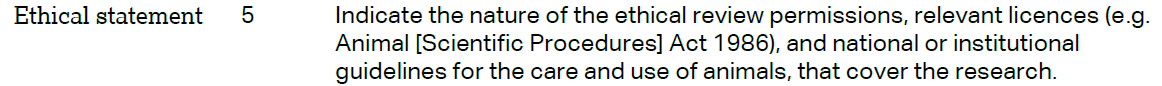 | | | Paragraph 1 |  |
| 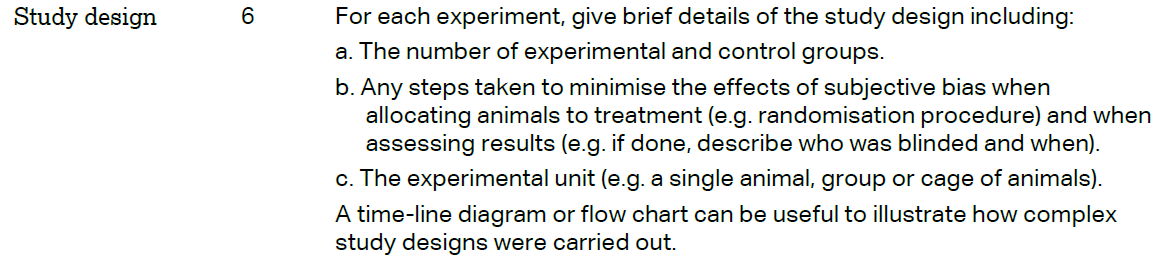 | | | Paragraph 1  Paragraphs 8-9  Paragraphs 6  Figure1 |  |
| 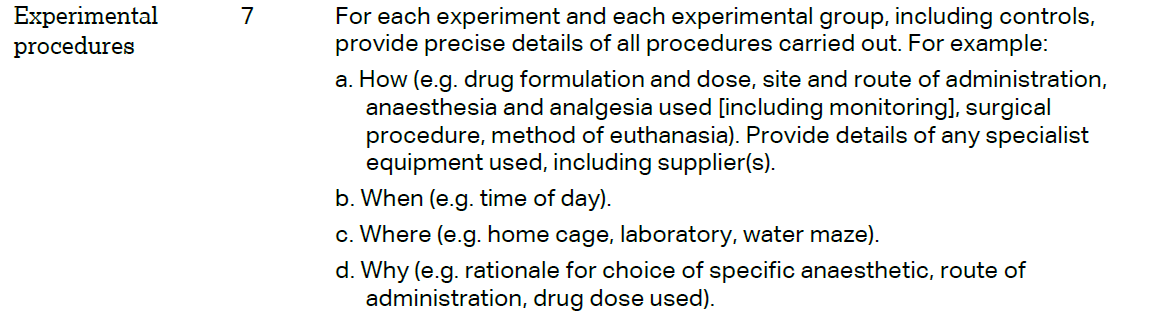 | | | Paragraphs 2-7,10-12 |  |
| 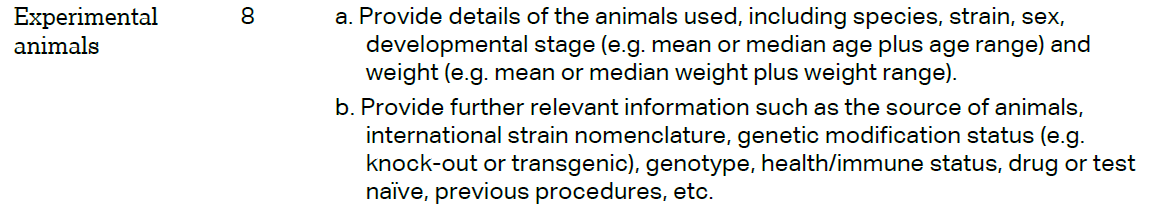 | | | Paragraph 1 |  |

| 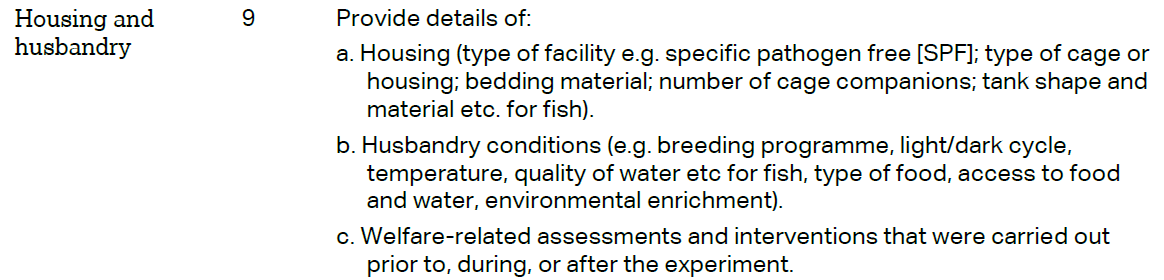 | Paragraph 1 | |
| --- | --- | --- |
| 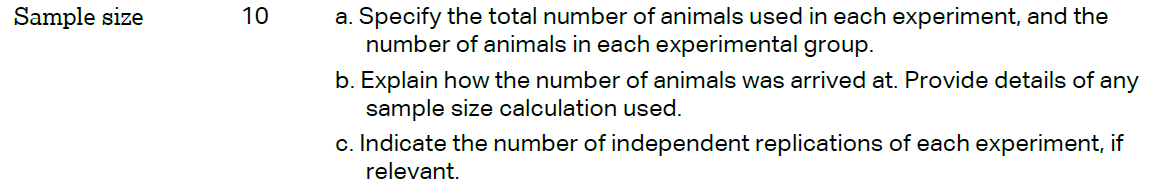 | Paragraph 1 | |
| 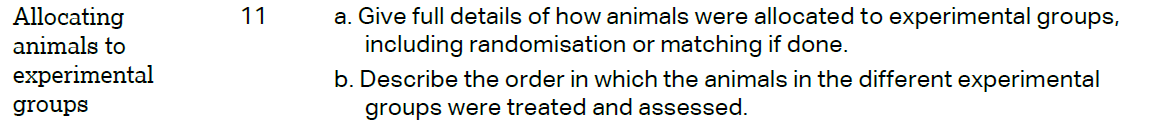 | Paragraph 9 | |
| 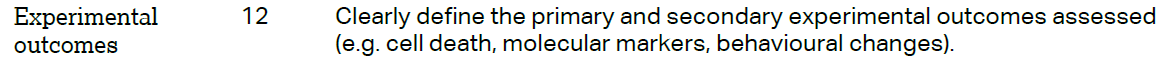 | Paragraph 13 | |
| 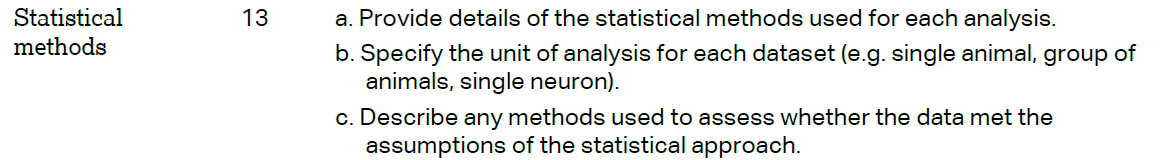 | Paragraphs 13&14andDiscussion  Paragraph  7 | |
| RESULTS |  | |
| 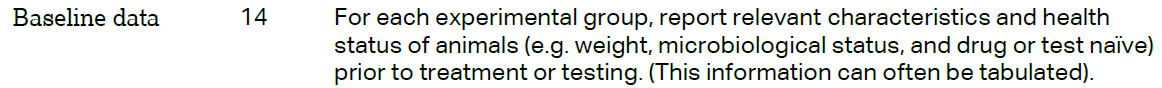 | Methods  Paragraph 1 | |
| 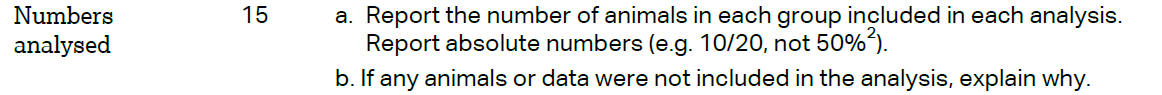 | Methods  Paragraph 1 | |
| 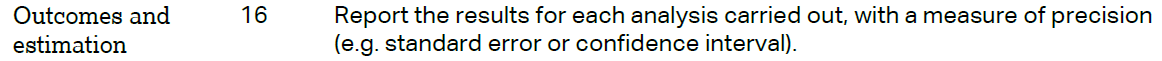 | Paragraphs 2 7 4 andFigures3&4 | |
| 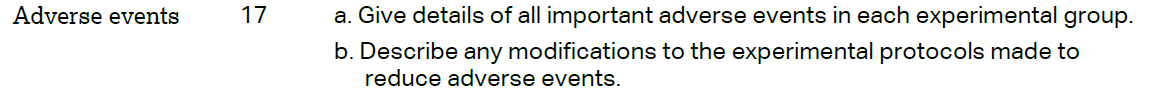 | Paragraph 1 | |
| DISCUSSION |  | |
| 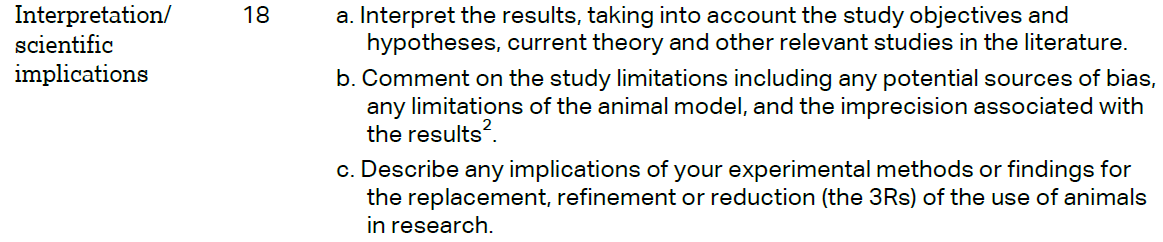 | Throughout  Paragraph 8  Paragraphs 8&9 | |
| 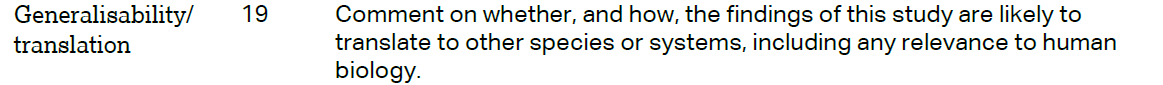 | Paragraphs 4&11 | |
| 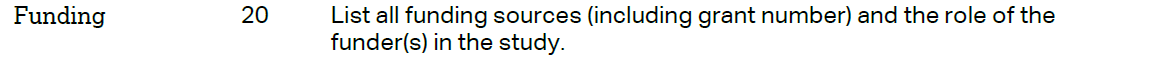 | | Paragraph 14 |


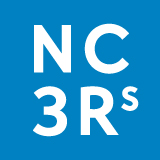

Supplement: S1 File — (DOCX) [file pone.0190515.s001.docx]
